# Bacterial extracellular vesicles and their novel therapeutic applications in health and cancer

**DOI:** 10.3389/fcimb.2022.962216

**Published:** 2022-11-11

**Authors:** Niloufar Hosseini-Giv, Alyza Basas, Chloe Hicks, Emad El-Omar, Fatima El-Assaad, Elham Hosseini-Beheshti

**Affiliations:** ^1^ Department of Biology, Faculty of Science, Ferdowsi University of Mashhad, Mashhad, Iran; ^2^ UNSW Microbiome Research Centre, St George and Sutherland Clinical Campuses, School of Clinical Medicine, Faculty of Medicine and Health, University of New South Wales, Sydney, NSW, Australia; ^3^ School of Medical Sciences, Faculty of Medicine and Health, University of Sydney, Camperdown, NSW, Australia; ^4^ The Sydney Nano Institute, The University of Sydney, Sydney, NSW, Australia

**Keywords:** bacterial extracellular vesicles, microvesicles, outer membrane vesicles, cell cargo, host interactions, infection, diagnosis, therapy

## Abstract

Bacterial cells communicate with host cells and other bacteria through the release of membrane vesicles known as bacterial extracellular vesicles (BEV). BEV are established mediators of intracellular signaling, stress tolerance, horizontal gene transfer, immune stimulation and pathogenicity. Both Gram-positive and Gram-negative bacteria produce extracellular vesicles through different mechanisms based on cell structure. BEV contain and transfer different types of cargo such as nucleic acids, proteins and lipids, which are used to interact with and affect host cells such as cytotoxicity and immunomodulation. The role of these membranous microvesicles in host communication, intra- and inter-species cell interaction and signaling, and contribution to various diseases have been well demonstrated. Due to their structure, these vesicles can be easily engineered to be utilized for clinical application, as shown with its role in vaccine therapy, and could be used as a diagnostic and cancer drug delivery tool in the future. However, like other novel therapeutic approaches, further investigation and standardization is imperative for BEV to become a routine vector or a conventional treatment method.

## 1 Introduction to bacterial extracellular vesicles

Adaptation and communication are critical factors for cell survival. The secretion of cytosolic compounds is essential for both intra- and inter-species cell interaction (Briaud and Carroll, 2020). All domains of life, including both prokaryotes and eukaryotes, produce membrane-derived lipid bilayer extracellular vesicles (EV) that have different cellular targets (Jahromi and Fuhrmann, 2021). Bacterial EV (BEV) are recognized as a type of bacterial secretion, and they are crucial mediators in intracellular signaling *via* the transfer of macromolecular cargoes (Jahromi and Fuhrmann, 2021). There are various subtypes of BEV known as membrane vesicles (MV), which are defined by their membrane origin and mode of release and have been described for both Gram-positive and Gram-negative bacteria (Nagakubo et al., 2019).

There are many accepted roles for BEV, including pathogenesis (Park et al., 2013), inter-species, intra-species, and inter-kingdom communication (Brown et al., 2015), stress tolerance (Kim et al., 2020b), horizontal gene transfer (Chronopoulos and Kalluri, 2020), and immune stimulation (Zhou et al., 2020).

BEV are nanoparticles that are stable in physiological conditions and have the capacity to transfer biomolecules and other cellular cargo. Due to such convenient properties of BEV, they could be utilized as a novel application for the treatment and diagnosis of disorders such as cancer and various infectious diseases (Jahromi and Fuhrmann, 2021). Despite the potential of engineering BEV for therapeutic goals, there are currently major limitations that should be addressed in future studies, one of which being a lack of standardized protocol for the purification and isolation of the various types of BEV and its cargo. Existing protocols for isolating BEV from human body fluids involve biophysical separation through methods of ultracentrifugation, ultrafiltration, and biochemical characterization such as size-exclusion chromatography (Tulkens et al., 2020). Working with body fluids is inherently challenging, and such BEV isolation methods are a complex and lengthy process that requires troubleshooting due to inconsistency in results, and would benefit from standardization in the future to improve the scientific rigor of BEV studies (Tulkens et al., 2020).

### 1.1 Biogenesis

**Figure 1:**
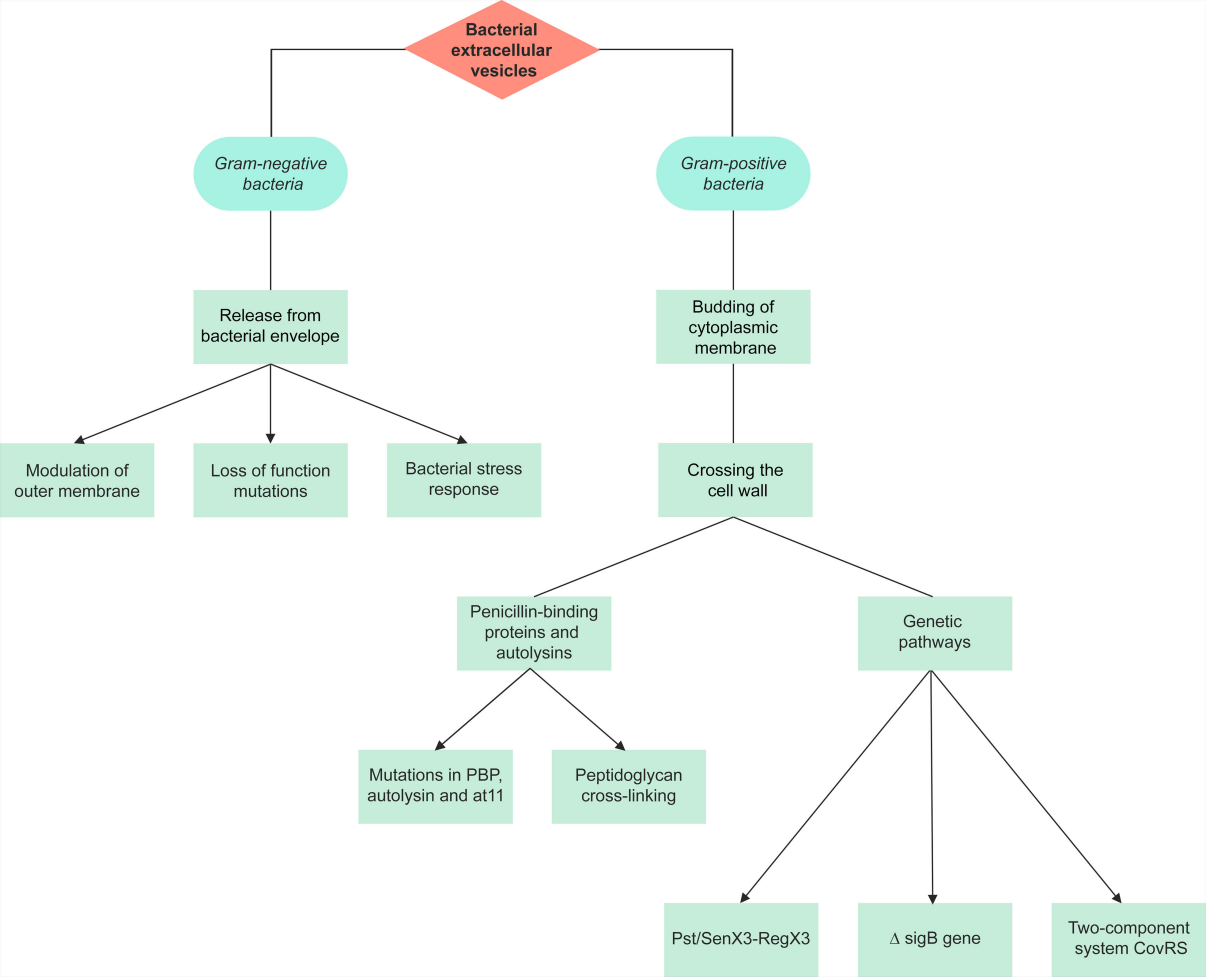
A summary of the pathways of extracellular vesicle biogenesis for Gram-negative and Gram-positive bacteria. The production of extracellular vesicles differs between Gram-negative and Gram-positive bacteria due to their different structures. There are 3 proposed models for the production of Gram-negative outer membrane vesicles, with the initial stage involving release from the bacterial envelope. Gram-positive bacteria lack an outer membrane, hence vesicles are formed through the budding of the cytoplasmic membrane and crossing of the cell wall. Gram-positive extracellular vesicles are synthesized either through penicillin-binding proteins and autolysins or genetic pathways.

Prokaryotic EV are divided into Gram-negative BEV and Gram-positive BEV (**Figure 1**). Prokaryotic EV are divided into distinct categories; outer-membrane vesicles (OMV), outer-inner membrane vesicles (O-IMV), cytoplasmic membrane vesicles (CMV) and tube-shaped membranous structures (TSMS) (Toyofuku et al., 2019). OMV are Gram-negative derived, hence the inner leaflet is made of phospholipid, and the outer leaflet consists of lipopolysaccharide (LPS), outer membrane proteins, and periplasmic proteins trapped during the budding of the membrane (Avila-Calderón et al., 2021). LPS, lipoproteins, outer membrane proteins, and flagellin are all molecules that have been proposed to contribute to OMV formation (Avila-Calderón et al., 2021). OMV cargo includes RNA, DNA, proteins, and virulence factors (Sjöström et al., 2015; Koeppen et al., 2016; Turnbull et al., 2016; Bitto et al., 2017). O-IMV are double-bilayer BEV originally described budding/blebbing from *Shewanella vesiculosa* (*S. vesiculosa*) cells, and specifically mediate the transfer of local intracellular components such as DNA during formation (Pérez-Cruz et al., 2013; Pérez-Cruz et al., 2015; Turnbull et al., 2016). Single and double-bilayer BEV can be formed *via* explosive cell lysis, in which cryptic prophage endolysin activity results in cell wall degradation or DNA damage giving rise to BEV formation (Turnbull et al., 2016; Toyofuku et al., 2017; Baeza et al., 2021).

Within MV, the proportion of O-IMV differs depending on the bacteria, ranging from 0.1% in *S. vesiculosa* M7-derived EV to 49% in *Pseudoalteromonas marina* (Pérez-Cruz et al., 2015). O-IMV were observed and quantified using transmission electron microscopy and cryo-transmission electron microscopy techniques (Pérez-Cruz et al., 2015). The third type, CMV, are released by Gram-positive bacteria (Toyofuku et al., 2019), though also seen in Gram-negative bacteria such as *Acidiphilium cryptum* JF-5 under stress conditions expressing vesicles derived from the cytoplasmic membrane (Küsel et al., 1999). In mycobacteria, it is proposed that its complex cell wall and surrounding capsule must undergo remodeling or degradation in order for MV to be freely released (Rodriguez and Prados-Rosales, 2016).

TSMS are the last type of BEV, and are known as nanotubes, nanowires, or nanopods. TSMS can be produced by both Gram- positive or Gram-negative bacteria and play a role in the component exchange by acting as a bridge between different cells (Toyofuku et al., 2019). TSMS have an average tube width of 50-70 nm and connect cells within biofilms at the periplasmic level, facilitating social activities (Baidya et al., 2018). *Myxococcus xanthus* produce OMV chains and TSMS that interconnect cells, which can allow for the intercellular transfer of molecules such as membrane protein (Remis et al., 2014).

#### 1.1.1 Gram-negative bacteria extracellular vesicles

It has been demonstrated that Gram-negative outer membrane vesicles (OMV) are released from the bacterial envelope (Volgers et al., 2018), which comprises of an inner glycerophospholipid bilayer membrane, a peptidoglycan layer, and an outer membrane (OM), with the periplasmic space separating the outer and inner membrane (Henderson et al., 2016). OMV are released for several purposes including host-microbe interactions, and their size varies between 20-400 nm (Schwechheimer and Kuehn, 2015). The interaction between host and microbes is mediated by OMV cargoes, including lipids, LPS, different types of proteins, small molecules, peptidoglycan, and nucleic acids. Moreover, the transfer of virulence factors as cargoes are mediated by OMV for the access to host tissues by Gram-negative bacteria (Jan, 2017). They can also affect the biofilm formation and host cell function modulations (Cecil et al., 2019).This type of EV has been studied in numerous Gram-negative bacterial strains such as *Escherichia coli* (*E. coli*), *Veillonella parvula*, *Vibrio cholerae* (*V. cholerae*)*, Salmonella enterica*, *Aeromonas* spp., *Brucella melitensis, Porphyromonas gingivalis* (*P. gingivalis*), and *Neisseria meningitidis* (*N. meningitidis*) (Zavan et al., 2020).

Three different models are suggested for OMV production (**Figure 1**): i) loss-of-function mutations result in deficient linkages between the OM and the periplasmic peptidoglycan layer, and the Tol-Pal complex, the periplasmic peptidoglycan layer and outer membrane proteins (Schwechheimer and Kuehn, 2013; Volgers et al., 2018). This model has been studied in *Salmonella enterica* serovar Typhimurium (*S.* Typhimurium), *Pseudomonas aeruginosa* (*P. aeruginosa*), *E. coli*, *V. cholerae*, *Acinetobacter baumannii* and *Haemophilus influenzae* (*H. influenzae*) (Deatherage et al., 2009; Godlewska et al., 2009; Wessel et al., 2013; Roier et al., 2016).

ii) bacterial stress responses and accumulation of envelope components. As of now, three primary stress responses have been found to play a role in the production of EV: a) the sigma factor E (σ^E^) response, b) the two-component regulator Cpx response, and c) the phage shock response (Volgers et al., 2018). This mechanism was also studied in *E. coli* and *P. aeruginosa* (Macdonald and Kuehn, 2013; Kim et al., 2015) iii) modulation of the OM: the OM’s stability is heavily dependent on LPS interactions, which can be affected by LPS structure and number, and other external and internal factors, subsequently affecting OMV production. Such factors include cationic antimicrobial peptides such as polymyxin B, cations such as Mg^2+^, LPS phenotype, changes in expression of LPS biogenesis pathway enzymes, temperature changes, pH, NaCl, phosphate and sucrose concentration, oxygen stress, and activation of other pathways that affect vesiculation (e.g. ABC transporter and Tol-Pol system) (Volgers et al., 2018).

#### 1.1.2 Gram-positive bacteria extracellular vesicles

Gram-positive BEV (CMV) range from 20-200 nm in diameter and are derived directly from the cytoplasmic membrane (Coelho et al., 2019). They were only recently recognized as it was previously assumed that Gram-positive bacteria could not release EV due to their thick peptidoglycan cell wall (Coelho et al., 2019). As such, investigations on CMV are very limited, and so mechanisms of their biogenesis are somewhat elusive.

The first stage of CMV production involves budding of the cytoplasmic membrane. Although the composition of the cytoplasmic membrane and CMV is similar, slight differences in their membranes have been reported, such as lipid components in *Bacillus anthracis* (*B. anthracis*) Sterne strain 34F2 bacterial cells (Rivera et al., 2010; Surve et al., 2016; Schlatterer et al., 2018). Based on this observation, it can be proposed that the CMV may undergo some changes within the cytoplasmic membrane. Some of these changes were studied in *Listeria monocytogenes* (*L. monocytogenes*) and *Streptococcus pyogenes* (*S. pyogenes*). Moreover, there may be species-specific mechanisms for the production of Gram-positive EV (Resch et al., 2016; Coelho et al., 2019; Briaud and Carroll, 2020).

The final stage of CMV production involves crossing through the cell wall. Penicillin-binding proteins (PBPs) and autolysins (cell wall proteins) were detected within some Gram-positive BEV, including *Staphylococcus aureus* (*S. aureus*), *B. anthracis*, *L. monocytogenes*, *S. pyogenes*, *Cutibacterium acnes* (formerly *Propionibacterium acnes*), *Filifactor alocis*, and Mycobacterium spp. (Lee et al., 2009; Rivera et al., 2010; Prados-Rosales et al., 2011; Lee et al., 2013; Resch et al., 2016; Jeon et al., 2017; Kim et al., 2020b). In *S. aureus*, the level of peptidoglycan cross-linking is proposed to play a role in EV release. Mutations in PBP, and autolysin were each shown to increase EV production, reduce EV size, and increase release respectively (Wang et al., 2018). BEV have been isolated from Gram-positive bacteria during different growth phases, therefore vesiculation can be assumed to be a continuous process (Kim et al., 2016b).

There are very few studies investigating the genetic pathways underlying Gram-positive CMV release. In *S. pyogenes*, *L. monocytogenes*, and *Mycobacterium tuberculosis* (*M. tuberculosis*), the inactivating mutation in the covS gene of the two-component responder/sensor gene regulatory system (CovRS), ΔsigB gene, and Pst/SenX3-RegX3 respectively have shown to play a role in the biogenesis of these BEV (Lee et al., 2013; Resch et al., 2016; White et al., 2018).

A complex gene network is also proven to be involved in Gram-positive CMV production, such as the deactivation of the *virR* gene which leads to hypervesiculation in *M.* tuberculosis (Rath et al., 2013). As aforementioned, further investigation is needed to understand the mechanisms involved in EV release by Gram-positive bacteria (Briaud and Carroll, 2020).

### 1.2 Cargoes

BEV are known to carry a variety of cellular cargo such as proteins, enzymes, DNA, RNA, peptidoglycan, and lipids (Chronopoulos and Kalluri, 2020; Ha et al., 2020).The capacity of BEV to transfer a wide variety of biological components makes them highly functional, and therefore they have become an area of great interest. Some of their functions include intra- and inter-kingdom communication, nutrient transfer amongst bacterial communities, host immune stimulation or suppression, and self-protection through the removal of unwanted proteins (Gao and Van Der Veen, 2020).

#### 1.2.1 Nucleic acids

##### 1.2.1.1 Ribonucleic acid (RNA)

RNA plays a role in biochemical reactions, gene expression regulation, destroying foreign genomes, and has high specificity for small molecules (Cech and Steitz, 2014). Extracellular RNA (exRNA) that affect recipient cells have been discovered in BEV; these messenger RNA (mRNA) and microRNA (miRNA) are transferred between cells *via* EV, and perform biological functions after entering host cells (Valadi et al., 2007). Bacterial RNAs have been detected in human plasma and as such, the immunostimulatory impacts of microbial RNAs on host cells and their molecular receptors have been studied for decades (Tsatsaronis et al., 2018).

The gut microbiome, including its composition, gene expression, growth, and shape, could be affected by miRNAs released by intestinal cells. However, recent evidence highlights that bacterial RNAs influence host cells (**Table 1**). Crosstalk between bacterial RNAs and host physiology was first studied in *Caenorhabditis elegans* (*C. elegans*). This study demonstrated that two *E. coli* endogenous non-coding small RNA (sRNA), OxyS and DsrA, could regulate the gene expression and physiology of *C. elegans* (Liu et al., 2012). These sRNA are expressed in *E. coli* under harsh environments and serve to protect the bacteria from overfeeding by *C. elegans* by activating or repressing gene expression (Liu et al., 2012).The gene suppression mediated by OxyS and DsrA resulted in impaired chemosensory behavior and decreased longevity in *C. elegans* (Liu et al., 2012). It was also proposed that *Bacillus mycoides* could also utilize non-coding RNA similar to OxyS to interfere with *C. elegans* gene expression and physiology (Liu et al., 2012). Various studies in eukaryotes have demonstrated that bacterial RNAs can alter other cells function and play a role in host immune response (Ghosal, 2017). However, it remains difficult to determine the exact composition of bacteria-derived RNAs, RNA trafficking, and mechanisms of host cell interaction (Ghosal, 2017). An RNA-binding protein, Hfq, which usually regulates the cytoplasmic mRNA translation by sRNA, was proposed to contribute to sRNA transfer (Malabirade et al., 2017) BEV have been proposed as a probable mechanism for intracellular RNA transport (Dauros-Singorenko et al., 2018).

**Table 1:** Studies on different types of RNA released by bacteria and their effects on host cells.

Bacterial species/strain	Type of RNA	Effect on host cell	Reference
***Aggregatibacter actinomycetemcomitans***	exRNAs	Crossing the blood brain barriers as well as delivery of EVs to brain monocytes and microglial interleukin (IL)-6 promotion. Inhibits IL-5, IL-13, and IL-15 secretion in Jurkat T-cells	(Choi et al., 2018; Ha et al., 2020)
***Borrelia burgdorferi***	mRNA sRNA	Not evaluated	(Malge et al., 2018)
***Escherichia*** ***coli***	EV-RNA	No cytotoxic or inflammatory effects on cultured bladder cells, but suppressed IL-1a response when combined with LPS treatments	(Dauros-Singorenko et al., 2020)
***Listeria monocytogenes***	Mainly rRNA	Transfected RNA or direct incubation of rli32 deletion strain EVs contribute to inhibition of IFN-B (macrophages)	(Frantz et al., 2019)
***Mycobacterium smegmatis***	Not evaluated	Not evaluated	(Dauros Singorenko et al., 2017)
***Neisseria gonorrhoeae***	Not evaluated	Not evaluated	(Dorward et al., 1989; Biller et al., 2014)
***Porphyromonas gingivalis***	16S rRNA, mRNAs (mfa1, sod, and fimA)	Inhibits IL-5, IL-13, and IL-15 secretion in Jurkat T-cells	(Ho et al., 2015; Choi et al., 2017b)
***Prochlorococcus marinus***	rRNA	Not evaluated	(Biller et al., 2014)
***Pseudomonas aeruginosa***	tRNA, ncRNA, mRNA, rRNA, tmRNA	LPS-induced IL-8 mRNA, and MAPK signaling pathway, and IL-8 secretion were inhibited by sRNA52320 in host epithelial cells and	(Turnbull et al., 2016; Koeppen et al., 2016)
***Salmonella enterica* serovar Typhimurium**	rRNA sRNA mRNA	Not evaluated	(Malabirade et al., 2018)
***Staphylococcus aureus* HG003**	mRNAs sRNAs tRNAs and rRNAs	Not determined	(Luz et al., 2021)
***Staphylococcus aureus* subspecies *aureus* Rosenbach MSSA476**	SsrA, RsaC, and RNAIII	Not determined	(Joshi et al., 2020)
***Streptococcus pyogenes***	rRNA tRNA	Not evaluated	(Resch et al., 2016)
***Streptococcus sanguinis***	msRNA	Not evaluated	(Choi et al., 2018)
***Treponema denticola***	msRNA	Inhibits IL-5, IL-13, and IL-15 secretion in Jurkat T-cells	(Choi et al., 2017b)
***Vibrio cholerae***	sRNA mRNA ncRNA	Not evaluated	(Sjöström et al., 2015)
***Vibrio fischeri***	sRNA SsrA	Upregulates genes coding for immune and antimicrobial activity, and reduced host cell robustness was observed	(Moriano-Gutierrez et al., 2020)

It was formerly believed that free RNA were unstable structures due to host Rnases. However, studies have shown that bacterial RNA can be stable outside of cells, with evidence of detection in many human body fluids (Chim et al., 2008; Becknell and Spencer, 2016). Furthermore, pathogenic RNA can form a secondary structure that is resistant to Rnases and it has been proven that RNA stability could be enhanced by binding to protective carriers. Moreover, RNA packaged in a membranous shield has led to protection against environmental factors and enzymes. BEV facilitate the targeted delivery of RNA into cells, fusion to the cell membrane, and the uptake of BEV cargoes (Benmoussa et al., 2016; Dauros-Singorenko et al., 2018). It was proposed that BEV uptake occurs at different rates by various host cells and therefore it can be conceived that each cell shows selective preferences or ability to uptake BEV (Vdovikova et al., 2017).

##### 1.2.1.2 Deoxyribonucleic acid (DNA)

It has been shown that in addition to RNA, EV from both prokaryotic and eukaryotic origins can harbor DNA. Horizontal gene transfer can be EV-based, occurring in both eukaryotes and prokaryotes. This DNA exchange directly impacts adaptation and evolution across species, stability and growth in different ecosystems and microbial populations, drug resistance, cooperation, and survival. Some limitations exist amongst known gene transfer mechanisms (transduction, transformation, and conjugation), including narrowed donor-recipient pairs in transduction, the small number of the possible natural recipients in transformation, and the limitation of genetic cargo amount in conjugation. Considering the massive interspecies gene transfer in the wild population, there is potential for another mechanism of gene transfer (Tran and Boedicker, 2017). There are some advantages to membrane-mediated DNA exchange, the most crucial factor being that released DNA remains intact when transported to the recipient cell. *Thermus thermophilus* release membrane-packaged DNAs, which are Dnase resistant and can target host cells, which can be helpful when targeting a specific microbial population or sub-population (Ben-Hur et al., 2019). The role of vesicular-DNA in cell-to-cell communication and environmental tolerance is yet to be fully understood. Certain bacterial species produce and release vesicular-DNA, which is impacted by external and developmental conditions (**Table 2**).

**Table 2:** External and developmental conditions impacting the production of vesicular-DNA by certain bacterial species.

**Bacterial species**	**External or developmental condition**	**Impact on vesicular-DNA production**	**Reference**
*Acinetobacter baumannii*	Carbapenem presence	Transferring drug resistant gene	(Chatterjee et al., 2017)
*Acinetobacter baylyi*	Antibiotic treatment	Increase	(Fulsundar et al., 2014)
*Helicobacter pylori*, *Pseudomonas aeruginosa*, *Salmonella enterica* serovar Ttyphimurium, *Porphyromonas gingivalis*	Exponential phase of bacterial growth	Packing into outer membrane vesicles	(Bitto et al., 2017)
*Proteus mirabilis*	Antibacterial agent	Vesicular cargo upgrade	(Ahmed et al., 2007)
*Pseudomonas aeruginosa*	Bacterial population reaches the threshold, Antibacterial drugs	Increase	(Allesen-Holm et al., 2006; Bitto et al., 2017)
*Streptococcus mutans*	During biofilm formation	Increase	(Liao et al., 2014)

Extracellular DNA (eDNA) was also studied in *Helicobacter pylori* (*H. pylori*), and *Pseudomonas putida* released during the biofilm phase. OMV aggregate in biofilm phenotypes, promoting its formation, and protecting *H. pylori* biofilms against Dnase I activity (Baumgarten et al., 2012; Grande et al., 2015). In *Lactobacillus reuteri* (*L. reuteri*), eDNA was found to be associated with both planktonic and biofilm phenotypes (Grande et al., 2017). A recent study used a flow cytometry technique to detect and quantify BEV eDNA-associated from *L. reuteri* and *H. pylori*, which suggests that BEV can be studied more effectively through flow cytometry (Puca et al., 2019). Plasmid transfer *via* BEV was also analyzed in *Buttiauxella agrestis*. In this study, naked plasmids were found to be degraded by Dnase I, while membrane-associated plasmids were stable and transferred to recipient cells successfully. Moreover, the naked plasmids could not transfer to host cells directly, therefore EV facilitated the transformation (Tashiro et al., 2017). However, Tran and Boedicker (2017) showed that plasmid identity could affect DNA packaging and transfer rates in *E. coli*, *Enterobacter cloacae*, and *Aeromonas veronii*. In addition, only genes with specialized sequences for gene trafficking were transferred, while *E. coli* DNA packaging did not depend on specialized sequences for gene transfer. The copy number of plasmids, their size, protein binding, location of each plasmid within the cell also impacted plasmid load into vesicles (Tran and Boedicker, 2017). In another study, chromosomal DNA packaging was investigated in *H. pylori*, *P. aeruginosa*, *S.* Typhimurium, and *P. gingivalis*. The packaging of the DNA into OMV was observed during the exponential phase of growth, with the majority of DNA located on the external surface of OMV and the remainder present in the vesicles (Bitto et al., 2017). Furthermore, in *H. influenzae* and *E. coli*, packaging DNA in MV for protection against degradation led to more uptake and genetic recombination (Vorkapic et al., 2016).

#### 1.2.2 Protein

BEV can transfer different types of proteins including bioactive proteins, lipoproteins, and virulence factors such as LPS and cytotoxic molecules to improve bacterial proliferation and survival in host cells (Chen et al., 2022). In addition to outer-membrane proteins, proteomic studies have suggested that inner-membrane and cytoplasmic components are present in OMV. The exact reason for their presence in OMV is still unknown.

##### 1.2.2.1 Proteins and pathogenicity

The selective trafficking of virulence factors is considered a favorable property for pathogens (Schwechheimer and Kuehn, 2015). *H. pylori* is an excellent example: in this bacterium, OMV lacked the VirD4 component of the type IV secretion system, therefore virulence factors were more easily secreted by parent cells. Moreover, these OMV contained the protease HtrA, which increases survival of bacterial cells in the presence of misfolded proteins (Schwechheimer and Kuehn, 2013; Olofsson et al., 2014). Interestingly, proteins that play a role in pathogen adhesion were excluded from OMV, therefore competition with the bacterial cell for host interaction was lost. For instance, in *H. pylori*, two adhesion factors that play a role in bacterial colonization, blood group antigen-binding adhesin and sialic acid-binding adhesin, are present in smaller amounts in OMV (Schwechheimer and Kuehn, 2015). Selective envelope proteins were also studied in *N. meningitidis*, which mainly contain autotransporter, iron, zinc uptake regulatory proteins, and two-partner secretion systems (Schwechheimer and Kuehn, 2015). BEV can include a wide variety of proteins and protein distribution can differ among species. In *S. aureus*, 90 different vesicular proteins have been identified, of which 56.7% are cytoplasmic proteins, 16.7% are membrane proteins, and 23.3% are extracellular proteins (Lee et al., 2009). This type of cargo can help recipient cells compete with other bacteria, confer antibiotic resistance, and aid virulence factors trafficking (Lee et al., 2009).

##### 1.2.2.2 Cargo sorting

It was shown that protein cargo sorting is a selective process in Gram-positive and Gram-negative bacteria (Bonnington and Kuehn, 2014; Brown et al., 2015; Tartaglia et al., 2020). It was believed initially that OMV components associate with the periplasm and OM, however it was found that OMV derived from *Serratia marcescens* and *P. gingivalis* did not contain any OM proteins and were instead enriched with several virulence factors or other proteins (McMahon et al., 2012; Veith et al., 2014). Gram-positive bacteria EV were enriched with lipoproteins. For example, *Bacillus subtilis* produces EV that contain lipoprotein and siderophore-binding proteins (Brown et al., 2014). *Mycobacterium bovis* and *M. tuberculosis* were demonstrated to release lipoprotein-enriched EV that can stimulate a Toll-like receptor (TLR) 2-dependent host response in mice (Prados-Rosales et al., 2011). EV derived from *B. anthracis* contain virulence factors specifically cytotoxic to macrophages, and *P. gingivalis* EV contain LPS and gingipains which results in the destruction of epithelial tissue once released (Chen et al., 2022). The protein components of *S. aureus* EV facilitate interbacterial protein transfer, antibiotic resistance and survival, and further biogenesis of EV (Lee et al., 2009).

Non-pathogenic BEV can also contain soluble proteins as a result of interaction with outer-membrane proteins or lipids. In *E. coli*, these soluble proteins were studied. When a carboxy-terminal is added to a soluble protein, OMV formation and chimeric soluble cargo are stimulated. This residue is known to trigger the σ^E^ heat-shock responses of the envelope (McBroom and Kuehn, 2007). The P_49_ protein secretion mechanism was studied in *S. vesiculosa* HM13 (Chen et al., 2019), which led to the proposal of a new strategy for cargo transport and delivery into BEV: that foreign proteins can transfer to BEV *via* fusion to P_49_ protein. Suggested applications for protein cargoes of BEV include invasion, adherence, antibiotic resistance, damage to host cells, impact on host immune response, biofilm formation, virulence promotion, and interspecies communication (Bonnington and Kuehn, 2014). Further investigation is required to determine the exact mechanism of protein sorting and trafficking by BEV.

#### 1.2.3 Lipids

One of the main components of BEV, alongside other biomolecules like DNA, RNA, proteins, and low molecular mass organic compounds, are lipids. Unlike other types of cargoes, limited research has been conducted on lipids in BEV. The most common lipid in Gram-negative EV are phosphoglycerolipids. Other lipids, including glycerolipids, lipoproteins, phospholipids, and LPS, were also found in lipidomic analyses of BEV. The lipid composition is completely different in Gram-positive bacteria compared to Gram-negative bacteria. For example, BEV derived from *S. pyogenes* are enriched in phosphatidylglycerol, and no cardiolipin was found in these BEV (Resch et al., 2016). BEV can exert immunomodulatory effects due to active biomolecules such as lipoproteins and LPS (Nagakubo et al., 2019). Lipids in OMV play a distinct role in their function. Some of these bacterial lipids, like LPS, can act as endotoxic agents. These toxic factors can be removed *via* engineering to make a safe vehicle for drug delivery. Moreover, the lipids of bilayer EV membranes can be adapted to the target environment and can be applied for targeted therapy (Yang et al., 2018).

## 2 BEV and host cells interactions

As previously mentioned, BEV help their cells of origin to communicate with and affect other cells (inter- and intra-kingdom). The mucosal-covered surfaces of hosts shelter both pathogenic and non-pathogenic bacterial species, so host-bacteria interactions were widely studied in these tissue types (**Table 3**). Toxin delivery into host cells occurs by BEV (Cecil et al., 2019).

**Table 3:** The impact of different EV cargoes on host bacteria.

Bacterial species	EV cargo	Impact on host	Reference
*Aggregatibacter actinomycetemcomitans*	sRNA	Cytokine production	(Choi et al., 2017b)
*Aggregatibacter actinomycetemcomitans*	RNA	Pro-inflammatory cytokine TNF-α *via* the TLR-8 and NF-κB signaling pathways in human macrophages	(Caruana and Walper, 2020)
*Campylobacter jejuni*	Cytolethal distending toxin	Cytotoxicity	(Lindmark et al., 2009)
*Escherichia coli*	Hemolysin, cytolethal distending toxin	Endothelial and epithelial apoptosis	(Bielaszewska et al., 2013; Kunsmann et al., 2015)
*Lactobacillus plantarum*	sRNA	Neuroinflammatory diseases development	(Choi et al., 2019)
*Porphyromonas gingivalis*	Gingipains, sRNA	Detachment or disrupt tight junctions of oral squamous epithelial cells, facilitating bacterial penetration, cytokine production	(Nakao et al., 2014; Choi et al., 2017b)
*Pseduomonas aeruginosa*	Alkaline phosphatase, β-lactamase, hemolytic phospholipase C and CFTR inhibitory factor, tRNA-Met derived sRNA	Biofilm formation, degradation of host antimicrobial peptides, cytotoxicity, and inhibition of chloride secretion in the airways, inhibit IR-8 secretion and MAP kinases expression	(Bomberger et al., 2009; Choi et al., 2011; Koeppen et al., 2016)
*Shigella dysenteriae*	Shiga toxin	Cytotoxicity	(Dutta et al., 2004)
*Treponema denticola*	Dentilisin, sRNA	Detachment or disrupt tight junctions of oral squamous epithelial cells, facilitating bacterial penetration, cytokine production	(Chi et al., 2003; Choi et al., 2017b)
*Vibrio cholerae*	Cholera toxin	Cytotoxicity	(Chatterjee and Chaudhuri, 2011)

### 2.1 Uptake of BEV by host cells

Although evidence suggests that BEV can enter host cells and change their physiology, the exact mechanism underlying their cargo sorting, uptake, and how they influence the host cells remains undetermined. Non-phagocytic cells uptake EV through five proposed mechanisms (**Table 4**): i) macropinocytosis, ii) clathrin-mediated endocytosis, iii) caveolin-mediated endocytosis, iv) lipid raft-mediated endocytosis, and v) direct membrane fusion (Caruana and Walper, 2020).

**Table 4:** The uptake of BEV by host cells through proposed mechanisms.

Proposed mechanism		BEV	Reference
**i)**	**Macropinocytosis**: Actin fibers are polymerized to form a ring under the cellular membrane and closes at the top of an enveloped extracellular vesicle	*Porphyromonas gingivalis* *Pseudomonas aeruginosa*	(Bomberger et al., 2009; Furuta et al., 2009)
**ii)**	**Clathrin-mediated endocytosis:** Clathrin-mediated vesicles form through a progression of protein complexes	*Aggregatibacter actinomycetemcomitans* *Brucella abortus* *Escherichia coli* *Helicobacter pylori*	(Caruana and Walper, 2020)
**iii)**	**Caveolin-mediated endocytosis:** Involves lipid raft membrane invaginations which are internalized depending on dynamin	*Escherichia coli* *Haemophilus influenzae* *Moraxella catarrhalis* *Vibrio cholerae*	(Mulcahy et al., 2014; Caruana and Walper, 2020)
**iv)**	**Lipid raft-mediated endocytosis:** Lipid rafts are more rigid and ordered, and are enriched in cholesterol and sphingolipids	*Acinetobacter baumannii* *Campylobacter jejuni* *Porphyromonas gingivalis* *Pseudomonas aeruginosa* *Vibrio cholerae* *Vibrio vulnificus*	(Mulcahy et al., 2014; Caruana and Walper, 2020)
**v)**	**Direct membrane fusion:** Direct fusion of EV with the host cell membrane, commonly at lipid raft domains	*Aggregatibacter actinomycetemcomitans* *Listeria monocytogenes* *Pseudomonas aeruginosa*	(Caruana and Walper, 2020)

Since the size and content of BEV are different from each other, a different way of endocytosis may be exploited as was discovered in *H. pylori* and *E.coli* (O'Donoghue et al., 2017; Zavan et al., 2019). Interaction of BEV with host cells can be affected by EV biogenesis mechanisms, the outcome of EV after host cell entry, and bacterial growth rate. Internalized BEV must not degrade and remain stable until the cargo has been delivered. However, this depends on the endocytosis mechanism; if the lysosomes are made after entering the host cells, they may degrade. The load is delivered to the endoplasmic reticulum/Golgi complex through the caveolin-mediated method, and then is transferred to the cytosol to affect the target cells (Caruana and Walper, 2020).

### 2.2 Immunomodulatory effects of commensal bacteria

Commensal bacteria can also affect host cells, specifically those that comprise the gut microbiome. These bacteria not only compete with pathogens, but also influence the production of bacteriocin, epithelial barrier function improvement, enzymatic activity, and short-chain fatty acids release, as well as impacting the host immune response in certain disease and leading to prevention or symptom inhibition (Plaza-Díaz et al., 2017; Molina-Tijeras et al., 2019). Although the gut lumen is covered with a mucus layer, the direct interaction between bacteria and host seems unlikely, therefore BEV must play a critical role in cargo exchange.

OMV released by the Gram-negative bacterium *Bacteroides fragilis* (*B. fragilis*) can stimulate dendritic cells in dextran sulfate sodium (DSS)-induced colitis, inducing a regulatory T cell (T_reg_) response, and can regulate autophagic genes to induce mucosal tolerance (Chu et al., 2016). Moreover, it can lead to anti-inflammatory stimulation and pro-inflammatory inhibition of cytokines in the Caco-2 cell line (Ahmadi Badi et al., 2019). OMV produced by *Bacteroides vulgatus* induce tolerance in bone marrow-derived colonic dendritic cells (Maerz et al., 2018). OMV derived from Gram-negative bacterium *E. coli* Nissle 1917 inhibit the DSS-induced colitis pro-inflammatory enzyme expression and enhance the tight junction protein expression, resulting in improved gut epithelial barrier function in colonic cell lines (Fábrega et al., 2017). Furthermore, *Akkermansia muciniphila* (*A. muciniphila*) OMV inhibit the progression of DSS-induced colitis. In high-fat diet obesity, these *A. muciniphila*-derived OMV stimulate the expression of tight junction proteins and enhance the integrity of the gut barrier (Chelakkot et al., 2018). In the context of cancer, in the CT26 mice colorectal cancer cell line, BEV induce pro-inflammatory cytokine production (Kang et al., 2013).

OMV derived from *P. aeruginosa*, which is a common pathogen for lung disease such as cystic fibrosis, were given to TLR2 and TLR4 knockout mice, resulting in increased pulmonary and alveolar macrophage concentrations of chemokines and cytokines, showing that OMV can induce pulmonary inflammation *in vivo* without the presence of live bacteria (Park et al., 2013).

The interaction between EV and host cells was also investigated in two Gram-positive probiotics genera, *Bifidobacterium* and *Lactobacillus* (Molina-Tijeras et al., 2019). *Lactobacillus sakei* EV strengthen the gut-blood barrier, positively affect microbiota composition, and stimulate IgA production (Yamasaki-Yashiki et al., 2019). EV released by *Lactobacillus rhamnosus* improve IL-10 output and increase the gut dendritic cells (Al-Nedawi et al., 2015). Pro-inflammatory cytokine production was inhibited in inflammatory bowel disease mouse models by EV derived from *Lactobacillus kefir*, *Lactobacillus kefiranofaceins*, and *Lactobacillus kefirgranum* (Seo et al., 2018). Additionally, a food allergy mouse model showed that *Bifidobacterium longum* EV suppress mast cell apoptosis, hence allergic diarrhea would be inhibited (Kim et al., 2016a). In another study, *Bifidobacterium bifidum*-derived EV stimulate a T_reg_ response through dendritic cells induction in naïve T cells (López et al., 2012). Other probiotic species that produce EV that induce dendritic cells and T_reg_ include *L. reuteri*, *Lactobacillus casei*, *Bifidobacterium animalis* and *Bifidobacterium adolescentis* (Smits et al., 2005; Baba et al., 2008 *)*. It was also proved that EV released by *Lactobacillus crispatus* and *Lactobacillus gasseri* can inhibit HIV-1 infection in some *ex vivo* models (Ñahui Palomino et al., 2019).

### 2.3 BEV and host cells during infection

BEV may play an important role in the progression and severity of bacterial infections due to their interactions with host cells. BEV are part of the immune evasion strategy of bacterial pathogens as they are released to resist host antimicrobial molecules. Various stressors to bacteria, such as antibiotics, and changes in temperature and pH, are known to cause changes in BEV production patterns to improve survival (Mozaheb and Mingeot-Leclercq, 2020). OMV induction in *E. coli* increases in response to sublethal concentrations of antimicrobial host defense peptides (HDP), which are a part of the innate immune system (Balhuizen et al., 2021). Peptides CATH-2 and PMAP-36, which interact with LPS and cause membrane permeabilization, were found mainly in the OMV rather than the *E. coli* bacterial pellet, suggesting that OMV are released to eliminate the parts of the membrane affected by HDP (Balhuizen et al., 2021).

During infection, changes in BEV formation have been demonstrated, particularly due to antibiotic treatment. In response to the antibiotic class aminoglycosides, which inhibit bacterial protein synthesis through binding on the 30S ribosome unit (Kotra et al., 2000), OMV formation is more likely to be induced and contain more DNA and cytoplasmic enzyme cargo (Kadurugamuwa et al., 1993). Exposure to gentamicin increased the number and size of OMV released from *Acinetobacter baylyi* increased, and the vesicles contained significantly more DNA cargo and efficient OMV-mediated plasmid DNA transfer (Fulsundar et al., 2014). Bacterial strains can increase their virulence potential through enzymes, prophages, and formation of biofilm. BEV may be key in transferring such virulence factors to host cells in response to antibiotics. Beta-lactamases, which are enzymes that break and inactivate beta-lactam antimicrobials, are packaged in OMV released by *P. aeruginosa* when exposed to benzylpenicillin (Ciofu et al., 2000). Treatment with ciprofloxacin enhanced the production of OMV and prophages in enterohemorrhagic *E. coli* (Bauwens et al., 2017), and increased both OMV number and the propensity for biofilm formation in *Francisella tularensis* (Siebert et al., 2019). In Gram-positive bacteria *Bacillus subtilis*, MV formation is greatly increased when the peptidoglycan cell wall is damaged by endolysins that are encoded by prophages (Toyofuku et al., 2017). Further investigations on the stressors and mechanisms conducive for BEV formation may be useful in production of BEV for future therapeutic applications and in the circumvention of further antimicrobial resistance.

## 3 Oncogenesis properties of BEV

There are very few studies of BEV in human body fluids due to technical challenges and other problems associated with their isolation from host-derived EV. BEV derived from the gut microbiome are assumed to take part in tumorigenesis of different types of cancer, including extra-gastric organs. They may indirectly promote cancer progression by inducing inflammation and forming a pre-metastatic niche (Chronopoulos and Kalluri, 2020). There is evidence to suggest that bacteria play a role in tumorigenesis. A recent mouse study demonstrated that when models of colitis-associated cancer are treated with antibiotics or are situated in a germ-free environment, there is an inhibition of tumor growth (Barteneva et al., 2017). Additionally, some bacterial species are known to play a role in intestinal cancers, including the *Mycobacterium avium* subspecies *paratuberculosis*, adherent enteroinvasive *E. coli*, and *Chlamydia pneumoniae* (Rubin et al., 2012). The microbiota composition and the number of some bacterial species change in patients with gastrointestinal tract cancers. *Enterococcus, Escherichia, Shigella, Klebsiella, Streptococcus, Peptostreptococcus, Firmicutes, Fusobacterium*, and *Bacteroidetes* were elevated in these cancers. They can affect normal cell proliferation and induce cancer proliferation by releasing toxins (for example, *B. fragilis* extracellular vesicles) or increasing inflammation and cancer cell proliferation (Barteneva et al., 2017). While it is evident that bacteria play a role in cancer development and progression, the mechanism underlying their role is unclear. This leads to the assumption that BEV are potential mediators of this interaction. It has also been shown that BEV associated with indoor dust may contribute to inflammation and diseases such as lung cancer by inducing neutrophilic inflammation through Th17 cell induction (Yang et al., 2020).

BEV derived from *H. pylori* are associated with the development of gastric cancer due to inflammatory mediator production by cells that uptake these EV. Such BEV contain the virulence factors cytotoxin-associated gene A and vacuolating cytotoxin A, which can induce the production of tumor necrosis factor-α, IL-6 and IL-1β by macrophages, and IL-8 by gastric epithelial cells. In mice, it was observed that these BEV could enhance the expression of interferon-gamma, IL-17, and EV-specific IgG. They also aggregate and remain in the stomach for a long period of time (Choi et al., 2017a).

Although some studies have shown the impact of BEV in different types of cancer, like other aspects of BEV, their exact role, and mechanism of action as a carcinogen agent remain unknown.

## 4 Application of BEV in diagnosis

It was recently proposed that BEV can play an essential role in diagnosis of some cancers. Based on Kim et al.’s (Kim et al., 2020a) analysis of BEV in relation to colorectal cancer (CRC), the gut microbiome was dramatically changed in participants with CRC, with *Firmicutes* and *Proteobacteria* being the most altered. They proposed that dysbiosis contributes to amino acid metabolism changes and CRC pathogenesis (Kim et al., 2020a).

BEV with active biological cargo can change their target cell function, and therefore have the capacity to induce asthma and chronic obstructive pulmonary disease. Lung cell-derived EV can play an important role in intercellular communication and assist with miRNA exchange. It was observed that serum BEV IgG antibody levels in patients were higher compared to healthy controls. Therefore, BEV could potentially be used as a diagnostic tool for lung disease (Kim et al., 2020c).

Another study performed by Riquelme et al. (2019) suggests that the microbiome of pancreatic adenocarcinoma (PDAC) patients can help predict survival rates. They found bacteria in the PDAC tumor site including *Pseudoxanthomonas*, *Streptomyces*, *Saccharopolyspora* and *Bacillus clausii*. This study found that the more bacteria within the tumor microenvironment (TME), the greater the expected survival rate (Riquelme et al., 2019). While this paper did not investigate BEV, it highlights the potential for their use in diagnosis and prognosis in PDAC.

OMV can be bioengineered for diagnosis using fluorescent techniques. Recently, the release of BEV from *A. muciniphila* were observed to enter and accumulate into bone tissues of osteoporotic mice, enhancing osteogenic activity and inhibiting the formation of osteoclasts (Liu et al., 2021). The OMV were labeled with the lipophilic fluorescent dye DiR iodide, and were discovered to be necessary for the bone protective effects of *A. muciniphila* (Liu et al., 2021). Measuring the levels of these OMV can theoretically be used to determine the efficacy of *A. muciniphila* replenishment in the correction of irregular bone metabolism and in protecting against osteoporosis.

## 5 Application of BEV in cancer therapy

BEV have been shown to play an important role in cancer therapy for drug delivery. Recently, EV derived from *Klebsiella pneumoniae* (*K. pneumoniae*) were studied by Kuerban et al. (2020), where they specifically prepared doxorubicin-loaded OMV (DOX-OMV). Based on their results, DOX can be delivered effectively to the lung cancer cell line NSCLC A549 cells, and inhibit tumor growth in mouse models. They have also proposed that OMV can recruit TME macrophages, enhancing DOX cytotoxicity *in vivo*. This novel idea enables the delivery of chemotherapeutic drugs while reducing off-target toxicity, triggering suitable immune responses and inhibiting cancer progression (Kuerban et al., 2020). OMV have been engineered to target specific cancer cells, particularly utilizing *E. coli*. To utilize BEV as transdermal nanoplatforms for melanoma therapy in mice, *E. coli*-derived OMV were modified with α_v_β_3_ integrin targeting ligand and indocyanine green (Peng et al., 2020). Upon topical application to the melanoma site, these OMV can significantly penetrate the stratum corneum and target melanoma cells with high specificity *via* binding between the α_v_β_3_ integrin and ligand on the cell surface. Furthermore, photothermal irradiation by near-infrared light, indocyanine green dissociates from the OMV and induces both peroxidase-antiperoxidase activity and hyperthermia, resulting in necrosis in tumor spheroids. This photothermal effect continues to deform the OMV and cause the release of tumor necrosis factor related apoptosis-inducing ligand, which then binds to death receptors in the cancer cell surface and activation of apoptosis. This combined photothermal and OMV treatment resulted in a 20- 50% increase of antiproliferation rates, and a 20-76% decrease in invasion capacity, in melanoma cell lines. Tumor relapse and metastasis is also prevented with this therapy through interfering specific genes and proteins, such as decreasing vimentin and increasing E-Cadherin levels. This approach is advantageous due to its safety and biocompatibility (Peng et al., 2020).

OMV released from mutated *E. coli* were used to transfer cell-specific anti-cancer siRNA, and it also displayed the human epidermal growth factor receptor 2-specific affibody. This siRNA targeted kinesin spindle protein and resulted in gene silencing. Hence, significant tumor growth regression was observed in the animal model of ovary adenocarcinoma and breast ductal cancer. These types of OMV did not show any immunogenic effects (Gujrati et al., 2014). Moreover, BEV can be used in TME reprogramming as an immune stimulation agent. However, OMV aggregation can lead to toxicity, and they may require an antibody to eliminate them whenever they are administrated intravenously. As such, Qing et al. (2020) covered the surface of *E. coli* BL21(DE3)-derived OMV with calcium phosphate. This reduced the risk of adverse effects with using these vesicles, and they were used for TME reprogramming. The shells were pH-sensitive, which helped to neutralize the acidic TME. It was also shown that OMV facilitated the M2-to-M1 polarization of macrophages and enhanced the antitumor effect. This method can also be applied in combination therapy and combined with other therapeutic agents (Qing et al., 2020).

In *E. coli* W3110-derived OMV, Kim et al. (2017) investigated their anti-cancer effect in colon cancer. The vesicles could specifically aggregate in the tumor site and induce long-term antitumor immune response, thus tumor regression was observed. Moreover, they also induced the production of CXCL10, an anti-cancer cytokine, and interferon-γ. They proposed that these OMV could be used as an immunotherapeutic agent in different types of cancer without any apparent side effects (Kim et al., 2017).

*E.coli*-derived OMV normally contain proinflammatory molecules such as LPS, inducing severe host innate immunity responses (Park et al., 2021). Therefore, synthetic BEV were produced from *E. coli* through a procedure involving the removal of periplasmic contents and disruption of cell membranes *via* incubation with lysozyme and sonication (Park et al., 2021). The bacterial inner membrane was then removed with suspension in an ionic detergent, and the resulting membrane was treated with high pH solution which then forms membrane sheets (Park et al., 2021). After ultracentrifugation, the purified sheets were collected and the synthetic BEV were isolated after a final sonication (Park et al., 2021). Even at higher doses than *E.coli*-derived OMV, synthetic BEV were shown to have no toxicity with no significant increases in TNF-α and IL-6 levels (Park et al., 2021). The naturally derived OMV and synthetic BEV were found to similarly activate dendritic cells derived from mouse bone marrow, and when combined with tumor tissue-derived EV, could induce melanoma tumour regression through contribution towards Th1-biased immune responses and enhanced cytotoxic T lymphocyte responses (Park et al., 2021). It is then expected that synthetic BEV can synergistically induce immunogenicity, however they likely require combination with other TLR ligands to sustainably generate potent tumour-specific cytotoxic T lymphocyte responses in humans (Park et al., 2021).

As many studies have proposed, the gut microbiota can affect distal tissues or organs, without any direct connection to the gut. The gut-lung axis is one prominent example. Bacteria and their products can translocate through the gastrointestinal barrier, such as with *Faecalibacterium prausnitzii*, which is a species with known anti-inflammatory effects. Jafari et al. (2019) used BEV on the A549 cancer cell line and evaluated the expression profile of cytokines and chemokines. These BEV significantly upregulated the anti-inflammatory cytokines, including IL-10, TGF-β2, and IL-1Ra, and downregulated some of the pro-inflammatory cytokines including IL-6, TNF-α, and TNF-β (Jafari et al., 2019).

BEV have application in cancer therapy as well as vaccine production *via* engineered bacteria. When modifying BEV endotoxicity in vaccine production, BEV are either treated with detergents to remove potential toxins such as LPS, or the originating bacteria is engineered to produce detoxified vesicles. In one study, *S. aureus* was engineered to produce detoxified cytolysins, and these BEV can induce an immune response and serve as a vaccine in a lethal sepsis model (Wang et al., 2018). BEV can also be utilized in producing vaccines for infectious diseases, such as Bexsero, an FDA-approved vaccine used to prevent meningococcal B disease (Zhang et al., 2019).

Moreover, a study on breast cancer microbiome showed that *K. pneumoniae*-derived EV were known to enhance anti-cancer effects of tamoxifen on breast cancer cell line (MCF-7) compared to cells were not treated with bacterial EV. These EV elevated the anti-hormonal effects of tamoxifen through *Cyclin* E2 and *p*-ERK, and cancer cell growth was inhibited. BEV may play an important role in breast cancer hormone therapy (An et al., 2021)

In addition, *Lacticaseibacillus rhamnosus* GG-derived EV were studied for their cytotoxic effects on hepatic cancer cell line HepG2. These EV significantly enhanced *bcl-2* and *bax* genes expression, and this overexpression led to the anti-proliferative effect of cancer cells and apoptosis (Behzadi et al., 2017).

**Figure 2:**
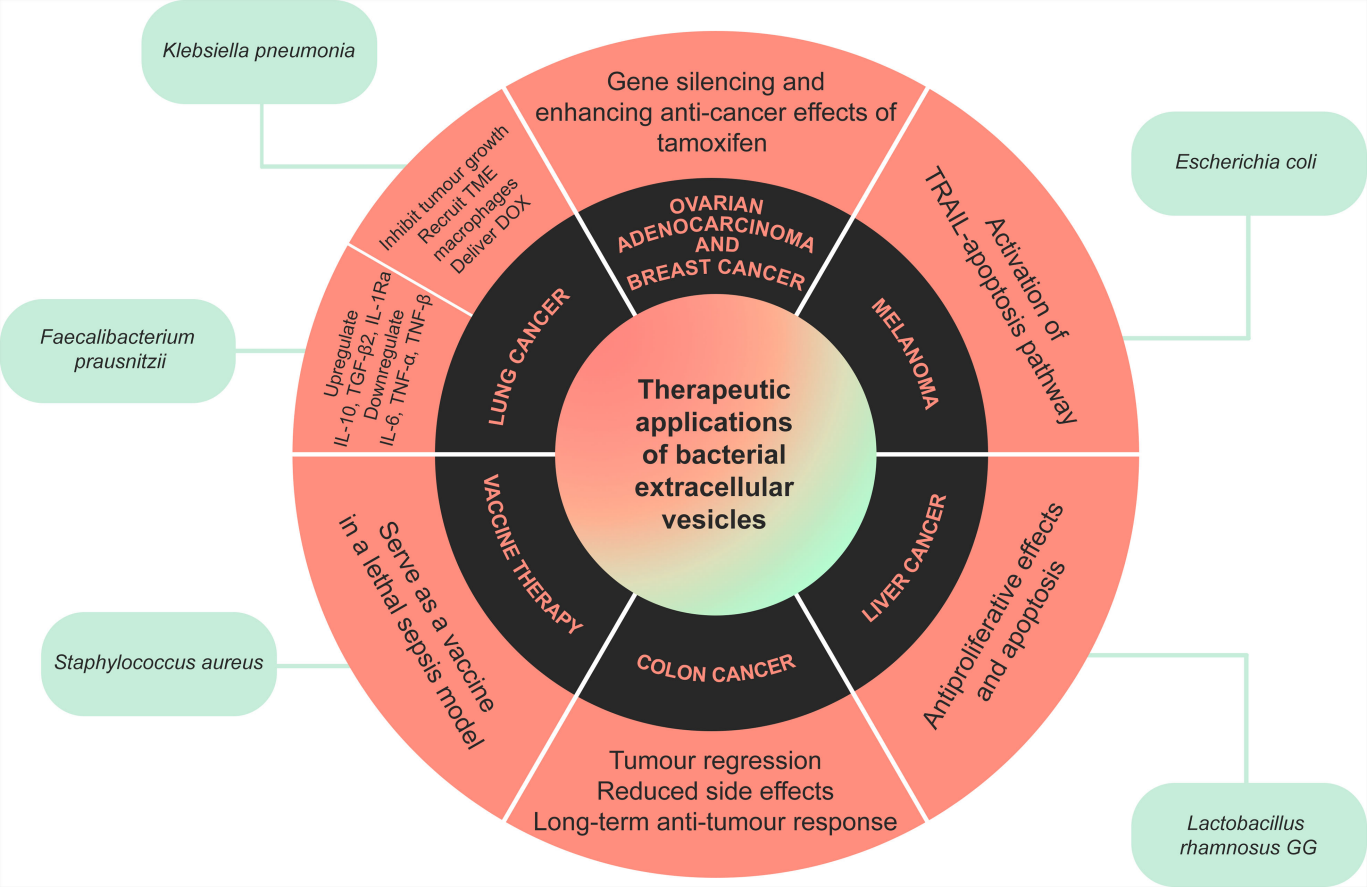
A summary of the therapeutic applications of bacterial extracellular vesicles (BEV). BEV released by certain bacteria have been shown to have beneficial anti-cancer and anti-inflammatory effects in cancers such as colon, breast, lung, and liver cancer, melanoma and ovarian adenocarcinoma. Extracellular vesicles released by *Staphylococcus aureus* can also trigger the immune system, serving as a vaccine in a model of lethal sepsis.

BEV evidently have the potential for clinical applications, including cancer therapy, vaccine development, infection control, and many other medical applications (**Figure 2**). BEV can be biocompatible and safe, and they can also be engineered to become properly suited for medical application. Although BEV has been investigated as a therapeutic option, like many other new treatments, this field requires further human and prospective studies.

## 6 Conclusion

The discovery of BEV and their functionality is paving a new way forward for novel diagnostic and therapeutic tools. BEV have the capacity to store and transport cellular cargo including DNA, RNA, proteins and lipids, while also being able to evade host degradation. These properties enable BEV to serve as important mediators in cellular communication. The BEV of commensal bacteria are essential in facilitating host-bacteria interactions and infection prevention, highlighting their beneficial impact on host health. Since these membranous nanoparticles can be engineered to perform specific interactions and functions, there is potential for BEV to be utilized as novel diagnostic and therapeutic tools. Certain BEV have the capacity to trigger immune responses, transport drugs, possess anti-cancer effects, or they may be associated with specific cancer types. As such, they can be used in cancer treatments, vaccine development or in diagnosis of some diseases. Despite the exciting potential of BEV, there are some limitations including the aggregation of EV or bacteriotoxin in the host, the high cost and complexity surrounding purification and separation of EV, low levels of protective antigen expression, and the interference of immunosuppressive molecules with the immune response.

Although BEV have great potential for use in the prognosis, diagnosis, and treatment of many types of diseases, there are some limitations to their application:

1) further research is needed as the exact mechanism of BEV biogenesis are still hypothesized. 2) purification and separation techniques require very time-consuming and complex laboratory procedures. 3) most studies on BEV are more related to vaccine development in comparison with other biomedical fields. 4) low levels of protective antigens expression and 5) BEV can carry cargo that can interfere with and suppress immune responses.

Due to their stability, size, lack of replicative ability and immunogenic cargo and properties from as the parent bacteria, BEV are promising candidates in playing a role in diagnosis, drug delivery, and disease therapy. From a therapeutic standpoint, BEV can be engineered to elicit immune responses in specific diseases such as infections or cancer. As seen in particular bacterial species, a potent T cell response can be induced and result in tumor regression. However, as with majority of the studies on BEV, such significant results are non-replicable in humans when solely using BEV. Further research on potential ligands and other synergistic molecules can improve the replicability and efficacy of BEV in human therapeutic applications. Further elucidation of BEV biogenesis, particularly in less-understood organisms such as Gram-positive bacteria, and their mechanisms of entry and pathogenicity in host cells, can help direct the engineering of BEV into a more efficient and applicable direction.

## Author contributions

NH-G and AB wrote the manuscript. CH, EE-O, FE-A, and EH-B revised the manuscript and provided guidance on the structure and content

## Acknowledgments

EH-B acknowledges the Dust Diseases Board competitive grant.

## Conflict of interest

The authors declare that the research was conducted in the absence of any commercial or financial relationships that could be construed as a potential conflict of interest.

## Publisher’s note

All claims expressed in this article are solely those of the authors and do not necessarily represent those of their affiliated organizations, or those of the publisher, the editors and the reviewers. Any product that may be evaluated in this article, or claim that may be made by its manufacturer, is not guaranteed or endorsed by the publisher.

## Author disclaimer

The views expressed herein are those of the authors and are not necessarily those of iCare or the Dust Diseases Board.

## Glossary

**Table:**

A. muciniphila	Akkermansia muciniphila
B. anthracis	Bacillus anthracis
B. fragilis	Bacteroides fragilis
BEV	Bacterial extracellular vesicles
bp	base pairs
C. elegans	Caenorhabditis elegans
C. jejuni	Campylobacter jejuni
CMV	Cytoplasmic membrane vesicles
CovRs	Control of virulence regulator sensor
CRC	Colorectal cancer
DNA	Deoxyribonucleic acid
DOX	Doxorubicin
DOX-OMV	Doxorubicin-loaded outer membrane vesicles
DSS	Dextran sulfate sodium
E. coli	Escherichia coli
eDNA	Extracellular DNA
EV	Extracellular vesicles
exRNA	Extracellular RNA
H. influenzae	Haemophilus influenzae
HDP	Host defense peptides
H. pylori	Helicobacter pylori
IL	Interleukin
K. pneumoniae	Klebsiella pneumoniae
L. monocytogenes	Listeria monocytogenes
L. reuteri	Lactobacillus reuteri
LPS	Lipopolysaccharide
M. tuberculosis	Mycobacterium tuberculosis
miRNA	Micro RNA
mRNA	Messenger RNA
MV	Membrane vesicles
N. meningitidis	Neisseria meningitidis
O-IMV	Outer-inner membrane vesicles
OM	Outer membrane
OMV	Outer membrane vesicles
P. aeruginosa	Pseudomonas aeruginosa
P. gingivalis	Porphyromonas gingivalis
PBPs	Penicillin-binding proteins
PDAC	Pancreatic adenocarcinoma
RNA	Ribonucleic acid
RNAse	Ribonuclease
S. aureus	Staphylococcus aureus
S. pyogenes	Streptococcus pyogenes
S. Typhimurium	Salmonella enterica serovar Typhimurium
S. vesiculosa	Shewanella vesiculosa
sRNA	Small RNA
TLR	Toll-like receptor
Treg	Regulatory T cell
TSMS	Tube-shaped membranous structures
V. cholerae	Vibrio cholerae
σE	sigma factor E
